# Effects of strength training on isometric muscle strength and muscle cross-sectional area in adults with a Fontan circulation: a pilot study

**DOI:** 10.1016/j.ijcchd.2026.100716

**Published:** 2026-09-27

**Authors:** Anna Wikner, Frida Bergman, Daniel Rinnström, Karna Johansson, Johan Ljungberg, Bengt Johansson, Camilla Sandberg

**Affiliations:** a Department of Public Health and Clinical Medicine, Umeå University, Umeå, Sweden; b Department of Diagnostics and Intervention, Umeå University, Umeå, Sweden; c Kiruna Research Unit, Umeå University, Kiruna, Sweden; d Department of Community Medicine and Rehabilitation, Umeå University, Umeå, Sweden

**Keywords:** Adult congenital heart disease, Total cavo-pulmonary connection, Muscle strength, Muscle mass, Muscle density, Physical activity

## Abstract

**Background:**

Adults with complex congenital heart disease, particularly those with a Fontan circulation, often have impaired muscle strength and reduced muscle mass from early age. Despite these concerns, data regarding the effects of strength training in this population is limited.

**Aim:**

To evaluate the impact of a 10-week strength training intervention on muscle strength and muscle cross-sectional area (CSA) in adults with Fontan circulation, compared to age- and sex matched controls.

**Methods:**

Nine adults with a Fontan circulation (median age 28.9 [IQR: 23.4-35.0], 4 women) underwent a gym-based strength training program. Isometric muscle strength (elbow flexion, knee extension, grip strength), radial and tibial muscle CSA and density, and habitual physical activity (accelerometer-based and self-reported data) were measured pre- and post-intervention. Within-group changes were analysed using the Wilcoxon signed-rank test; between-group differences were assessed using the Mann Whitney *U* test.

**Results:**

Both patients and controls demonstrated improvements in isometric elbow flexion strength (Patients: +6.2% [IQR: 2.8-17.7], *p* = 0.02, r = 0.77; Controls: +8.4% [IQR: 2.9-16.0], *p* = 0.012, r = 0.84) and tibial cross-sectional area (Patients: +5.0% [IQR: 2.7-11.4], *p* = 0.011, r = 0.85; Controls: +4.0% [IQR: 1.4-7.4], *p* = 0.018, r = 0.89). No changes were observed in isometric knee extension or grip strength. There were no differences between groups, and no adverse events occurred.

**Conclusion:**

This pilot study supports that strength training is safe and effective in improving muscle strength and muscle CSA in adults with a Fontan circulation. Despite the small sample size these findings are reassuring. However, confirmation in larger prospective trials is warranted.

**Clinicaltrials.gov, ID:**

NCT05454254, https://clinicaltrials.gov.

## Highlights

• Strength training increases upper-limb strength in adults with Fontan circulation.
• Lower-limb muscle size improved following the training program.
• Gains were comparable to responses in healthy controls.
• No adverse events were observed during the intervention.
• Strength training safely enhances muscle function in adults with Fontan circulation.

## Graphical abstract

**Figure:**
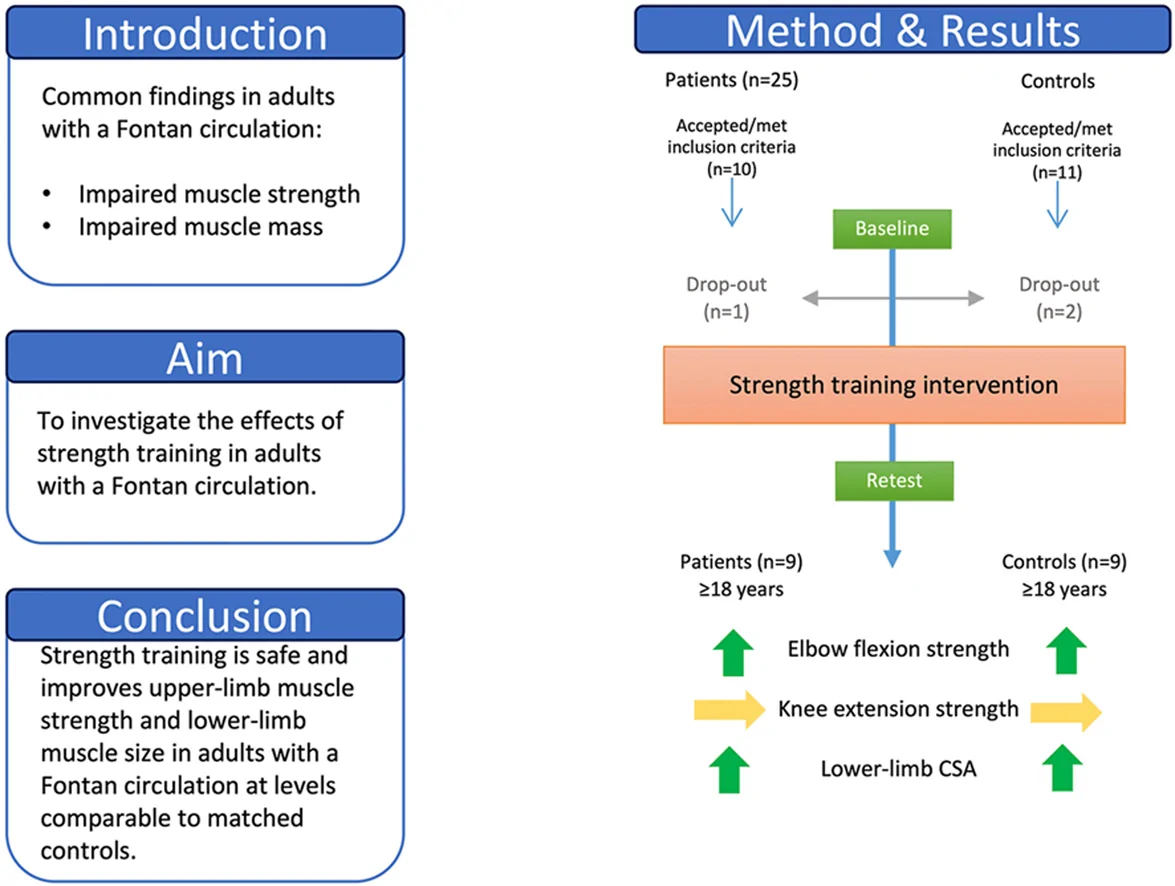

## 1 Introduction

Generalised impaired muscle strength and muscle mass are prevalent among adults with complex congenital heart disease (CHD) [1,2], with particularly pronounced deficits observed in individuals with a Fontan circulation [3,4]. These impairments tend to emerge at a young age, thus affecting the patients for most of their lives [5]. Individuals with a Fontan circulation have a unique circulation physiology characterised by the absence of a subpulmonary ventricle. As a result, they depend on the peripheral skeletal muscle pump to support venous return, especially during physical exertion [6]. Adequate venous return is essential for augmenting ventricular preload and thereby also cardiac output. When preload and cardiac output is limited, this leads to reduced exercise capacity, a common finding in this population. Consequently, strength training may be especially beneficial for individuals with a Fontan circulation, given their dependence on skeletal muscle function to support cardiovascular performance.

Although physical activity is strongly associated with muscle function, previous studies have shown that adults with CHD are as physically active as matched controls [7]. This suggests that physical inactivity alone does not fully explain the observed impairments in muscle function. Beyond activity-related factors, other potential contributors to impaired muscle function include cyanosis prior to surgical palliation and the unique cardiovascular pathophysiology associated with Fontan circulation [8].

A previous study involving adults with complex CHD, of whom 30% had a Fontan circulation, demonstrated reduced isometric muscle strength and muscle mass in both upper and lower limbs compared to controls. Importantly, patients and controls had a similar correlation between muscle strength and muscle mass, indicating that the ratio of produced muscle force per unit of muscle mass was preserved [9]. These findings indicate that skeletal muscle efficiency remains intact, and that reduced muscle strength is primarily attributable to reduced muscle mass. Therefore, strength training interventions ought to have positive effects on muscle strength and muscle mass.

However, evidence regarding the effects of strength training on muscle function in this population, especially among adults, is limited [10]. One small study (*n* = 11; training group *n* = 6, control group *n* = 5) reported improvements in peak oxygen uptake and cardiac output following strength training. Additionally, increases in calf muscle mass and combined measures of muscle strength were observed [11]. Similarly, a randomised controlled trial in children with a Fontan circulation reported improvements in peak oxygen uptake, stroke volume and isometric muscle strength after strength training. A third study reported improvements in work rate but unchanged peak oxygen uptake in adolescents with a Fontan circulation following strength training [12]. The interventions were however focused on the lower extremities and had primary outcomes other than muscle strength.

We have previously shown that strength training improves dynamic muscle strength in both upper and lower extremities in adults with a Fontan circulation [13]. However, the specific effects of strength training on isometric muscle strength and muscle composition in adults with a Fontan circulation remain underexplored.

Therefore, the primary aim of this study was to investigate the effects of strength training on isometric muscle strength in adults with a Fontan circulation, compared to matched controls. Secondary aims included the effects of strength training on i) muscle cross-sectional area, ii) muscle density iii) self-reported physical activity levels, and iv) accelerometer-measured physical activity levels.

## 2 Material and methods

### 2.1 Study population

This pilot investigation was conducted as a non-randomised controlled trial, comprising a 10-week strength training intervention aimed at enhancing dynamic muscular function (Graphical abstract). Adults with a Fontan circulation were enrolled between August 1, 2022, to August 31, 2023, from the Northern Healthcare Region of Sweden. Patients were included if aged 18 years or older with clinically stable Fontan circulation. Exclusion criteria encompassed engagement in regular strength training (≥2 sessions/week), comorbidities potentially affecting physical activity (*e.g*., rheumatoid arthritis), cognitive or neurological impairments, or other limiting conditions such as pregnancy.

Age- (±5 years, minimum 18 years) and sex matched control participants were recruited through convenience sampling from the catchment area of the study centre during the same recruitment period. The same exclusion criteria applied to controls, with the additional requirement of no known CHD. In total, 10 patients and 11 controls met inclusion criteria and accepted participation. All participants who completed the intervention (patients n = 9 and controls n = 9) were included in the final analysis (Graphical abstract). Selected results from this investigation have previously been published, alongside a detailed overview of the inclusion process and study design [13]. Written informed consent was obtained from all participants prior to enrolment. The study was approved by the Swedish Ethical Review Authority (2022-00058-01, 2022-04769-02).

### 2.2 Characteristics of participants

Diagnostic and surgical history data were obtained from the Swedish Registry of Congenital Heart Disease (SWEDCON). Additionally, baseline assessments included measurements of height, weight, waist and hip circumference, peripheral oxygen saturation, and current pharmacological treatment.

### 2.3 Isometric muscle strength

Isometric muscle strength in elbow flexion and knee extension was measured using a load cell (VZ101BH 500 kg, Anyload Weigh & Measure Inc.,NJ, US).

For elbow flexion, participants were instructed to grasp a handle with their dominant hand, maintain a supinated forearm (palm facing upward), with the elbow joint fixed at a 90° angle. They then performed a maximal isometric contraction of the elbow flexors, sustaining the effort for 5 s.

For knee extension, participants were seated on a bed with the feet suspended above the floor. The dominant lower leg was secured at a 90° knee flexion using a sling positioned just proximal to the medial and lateral malleoli. Participants were instructed to perform a maximal isometric contraction of the knee extensors and maintain the contraction for 5 s.

Grip strength was assessed using a hand dynamometer (JAMAR, Lafayette Instrument Co, IN, USA). Participants stood upright, held the dynamometer in their dominant hand with the elbow fixed at 90°, and performed a maximal grip contraction for 3 s.

Each test was repeated three times with a 1-min rest interval each repetition. The highest value recorded across the three tests was used for analysis.

### 2.4 Muscle cross-sectional area and muscle density

Muscle cross-sectional area (CSA) and density were assessed using peripheral quantitative computed tomography (pQCT, Stratec XCT2000, Medizintechnik GmbH, Pforzheim, Germany) at *Livsmedicin*, Umeå University Hospital, Sweden. This non-invasive imaging modality provides three-dimensional evaluation of peripheral soft tissue microarchitecture and composition including muscle and adipose tissue, at strictly defined points of the forearm or the lower leg.

Prior to each examination, the equipment was calibrated, and scans were performed with a voxel size of 0.5 x 0.5 × 2.0 mm and a slice thickness of 2 mm. The lengths of both the forearm and the lower leg were measured manually using a tape measure. Scans were performed at 4% (distal site) and 66% (proximal site) of the total bone length, measured from the distal end of the radius and tibia, respectively. An illustrative description of these anatomical measurement sites has been published previously [14]. Quantitative assessments of muscle and adipose tissue were obtained from the 66% site. At these measurement sites, the scans encompass all muscle compartments of the forearm and lower leg. To maintain consistency with the nomenclature used by the pQCT system, these measurement sites are hereafter referred to as the radial (radius) and tibial (tibia) sites.

The primary variables of interests were muscle cross-sectional area and muscle density. Muscle density serves as an indicator of muscle quality, with lower density values reflecting increased lipid infiltration within the muscle, whereas higher density corresponds to reduced intramuscular lipid content [15,16]. Importantly, higher muscle density has been associated with greater muscle strength, independent of muscle cross-sectional area, and is considered a marker of superior muscle quality [17].

By enrolment of the last two participants, the pQCT equipment was no longer available. Therefore, these two controls (women) did not undergo assessment of soft tissue before or after the intervention and were excluded from these analyses.

### 2.5 Habitual physical activity

#### 2.5.1 Self-reported data

Self-reported habitual physical activity was assessed at baseline and post-intervention using the short form of the International Physical Activity Questionnaire (IPAQ). The questionnaire captured data from the seven days preceding each assessment, specifically the week prior to baseline testing and the week prior to retesting. The questionnaire comprises four generic items evaluating time spent at different intensity levels of physical activity: vigorous, moderate, walking, and sedentary behaviour, during the past seven days. Responses were converted into a continuous score expressed as metabolic equivalents of task (MET) minutes per week, with vigorous activity assigned 8 METs, moderate activity 4 METs, and walking 3.3 METs. A total score of ≥3000 MET minutes per week was considered as high physical activity level; ≥600 MET minutes per week moderate; and <600 MET minutes as low physical activity level [18]. IPAQ has been validated in multiple countries, including Sweden [19].

#### 2.5.2 Accelerometer-based

Accelerometer-based physical activity data was assessed using the activPAL3 device (PAL Technologies Ltd, Glasgow, UK). Participants wore the monitor on the anterior right thigh for seven consecutive days following both baseline and retesting. Data were processed using ActiPASS version 2024.06.4 [[20], [21], [22]]. In accordance with proPASS criteria [23], days were excluded if they contained < 20 h of wear time/day, < 30 s of walking and > 30 min of activity classified as “other”, or ≥ 2 consecutive days without a valid sleep-interval. Based on these criteria, 15 of 236 monitoring days were excluded due to insufficient wear time/day. A manual quality check identified additional anomalies, including two days with no recorded walking, which were excluded. Furthermore, one day lacking a clear sleep interval and four days with unrealistically low activity, were flagged for a sensitivity analysis.

The primary variables included; 1) total daily step count; 2) the sleep interval, defined as the duration between first sleep onset and final awakening within each “time-in-bed” episode; 3) time spent in moderate-to-vigorous physical activity, defined as walking with cadence of 100-135 steps/min, or running, cycling, or walking at > 135 steps/min), 4) time spent in light-intensity ambulatory activity, defines as walking or slow movements at < 100 steps/min; and 5) sedentary behaviour, defined as time spent sitting or lying. One control participant lost the device after the intervention, and data from one patient could not be retrieved due to download failure, resulting in retest data for eight patients and eight controls.

The sensitivity analysis compared mean values of all physical activity variables between patients and controls with and without these five anomalous days. As their exclusion did not affect statistical significance, they were retained in the final analysis.

### 2.6 Intervention protocol

The intervention has previously been described in detail [13]. Briefly, it covered a 10-week period and involved strength training sessions conducted three times per week at a gym selected by each participant (Graphical abstract). The resistance regimen targeted major muscle groups in both upper and lower body and included exercises such as elbow flexion and extension, shoulder flexion, rowing, knee extension and flexion, heel raises, and deep squats. Additionally, grip strength was trained using an adjustable hand gripper. Training intensity (load) was tailored to the individual aiming for 10-12 repetitions per set across three sets. Through the intervention the load was progressed according to the principle of progressive overload, that is maintaining the 10-12 repetitions per set.

The initial training session was supervised by a study coordinator and conducted at the participant's chosen gym. All participants received access to a digital application (Exorlive, ExorLive AS, Oslo, NO), that provided the individualised training protocols. After each session, the participants completed a training log, documenting the sessions date, any modifications to load, and the number of repetitions and sets performed. In case of absence lasting seven days or more due to illness or other reason, the intervention period was extended by one week.

### 2.7 Statistical analysis

Statistical analysis was performed using IBM SPSS statistics (version 28.0.1.1, Armonk, NY, USA) and figures using GraphPad Software (version 10.6.1, LLC, San Diego, CA, USA). Due to the small sample, Mann Whitney *U* test was used for comparison of continuous variables between groups and presented as the median (IQR). For comparison of categorical variables, the chi-square test was applied. Within-group analyses were performed using the Wilcoxon signed rank test. Due to the small sample size, exact two-tailed p-values were used. Effect size estimates for non-parametric data were calculated as r = z/($\sqrt{N}$) with z being standardised test statistic and N equals number of participants in the equation. An effect size (r) was considered large if r > 0.5, medium if r > 0.3 and small if r > 0.1 [24]. The null hypothesis was rejected on *p* values < 0.05.

## 3 Results

### 3.1 Study population

Nine patients (28.9 [23.4-35.0] years, four women) and nine age- and sex matched controls were included in analysis. Age, sex, height, BMI, and waist/hip-ratio did not differ between patients and controls (Table 1). Patients had a lower oxygen saturation at rest compared to controls (92.0% [88.0, 95.0] vs. 98.0% [97.0, 98.5], *p* = 0.003). Cardiovascular pharmacological treatment was only present among patients, with detailed information published elsewhere [13]. The most common lesion among patients were tricuspid atresia (Table 2).

**Table 1:** Background characteristics.

Variable	All (n = 18)	Patients (n = 9)	Control (n = 9)	*p*
				
Age *years*	26.1 (24.5-31.9)	28.9 (23.4-35.0)	25.9 (24.8-31.4)	0.9
Sex *woman n (%)*	8 (44.4)	4 (44.4)	4 (44.4)	1.0
Height *cm*	172.4 (167.8-181.3)	171.8 (162.3-180.0)	178.0 (170.3-181.5)	0.3
BMI *kg/m*^*2*^	25.4 (22.5-26.8)	23.8 (21.8-27.7)	25.9 (23.9-27.4)	0.5
Waist/Hip *ratio*	0.87 (0.78-0.90)	0.88 (0.83-0.90)	0.83 (0.77-0.93)	0.7

Values are presented as *n* (%) or as median (25th-75th percentile). **Bold** values denote *p* < 0.05 for comparison between patients and controls.

BMI, body mass index.

**Table 2:** Patient characteristics and diagnoses.

Variable	Patients (n = 9)
Diagnosis	
HLHS	2 (22.2)
DILV	2 (22.2)
Tricuspid atresia	3 (33.3)
Functional single ventricle^†^	1 (11.1)
Pulmonary atresia	1 (11.1)
Age at TCPC *years*	3.8 (1.8, 10.4)

Values are presented as *n* (%) or as median (25th-75th quartile). ^†^ Unspecified.

HLHS, hypoplastic left heart syndrome; DILV, double inlet left ventricle; TCPC, total cavopulmonary connection.

As previously reported, the introduction to the intervention protocol took place at a mean time of 9 (IQR: 8-12) days following baseline testing, and participants were called back for retesting after a mean time of 13 (IQR: 12-14) weeks from baseline testing. The number of performed training sessions did not differ between patients and controls (23.0 [14.5-28.5] vs. 27.0 [23.5-29.0], *p* = 0.3). Post exertional exhaustion was reported by one patient, and difficulties in scheduling the training sessions were reported by another patient. The patients were able to complete the training protocol without any adverse events reported.

### 3.2 Isometric muscle strength

At baseline, patients and controls had similar isometric muscle strength in elbow flexion, knee extension and grip strength (Table 3).

**Tabell 3:** Isometric muscle strength, muscle mass, and muscle density before and after the intervention.

Muscle strength *N*	Patients (n = 9)					Controls (n = 9)					Patients vs. controls	
	*Pre*	*Post*	%	*p*	Cohens’D	*Pre*	*Post*	%	*p*	*Cohens’D*	*p*_baseline_	*p*%
Elbow flexion	127.5 (114.5-196.0)	173.5 (121.3-204.8)	6.2 (2.8-17.7)	**0.02**	*0.*771	172.0 (125.0-203.5)	184.0 (140-220.5)	8.4 (2.9-16.0)	**0.008**	0.841	0.4	0.86
Knee extension	339.5 (311.5-428.0)	352.5 (322.0-462.5)	5.5 (−3.0-11.6)	0.25	NA	456.5 (362.3-520.0)	461.5 (370.5-566.0)	6.9 (−2.0-12.7)	0.07	NA	0.2	0.86
Grip strength	34.0 (29.0-55.5)	34.0 (32.0-59.0)	6.7 (0-12.7)	0.14	NA	46.0 (33.0-49.5)	44.0 (34.0-62.0)	14.3 (−5.4-27.7)	0.055	NA	0.6	0.34

	Patients (n = 9)					Controls (n = 7)					Patients vs. control	
pQCT	*Pre*	*Post*	%	*p*		*Pre*	*Post*	%	*p*		*p*_baseline_	*p*%

RADIUS												
Muscle CSA *cm*^*2*^	28.5 (23.5-35. 6)	29.3 (25.1-36.7)	3.9 (0.0-6.0)	0.074	NA	36.2 (25.5-42.1)	39.0 (25.8-42.5)	3.0 (−1.6-6.0)	0.375	NA	0.2	0.6
Muscle density *mg/cm*^*3*^	76.5 (74.4-78.0)	78.3 (77.4-79.2)	1.9 (0.7-4.7)	**0.039**	0.691	76.6 (75.3-78.0)	77.2 (77.1-78.3)	0.8 (0.3-1.6)	0.063	NA	0.8	0.3
TIBIA												
Muscle CSA *cm*^*2*^	65.3 (58.6-71.1)	72.7 (59.3-74.6)	5.0 (2.7-11.4)	**0.008**	0.849	74.6 (52.8-83.0)	75.4 (53.6-89.9)	4.0 (1.4-7.4)	**0.016**	0.894	0.4	0.6
Muscle density *mg/cm*^3^	74.4 (72.6-76.0)	75.0 (73.2-77.5)	0.9 (0.0-1.7)	**0.039**	0.691	74.8 (73.7-76.3)	75.3 (74.5-76.8)	1.1 (0.6-1.6)	0.078	NA	0.6	0.8

Changes in isometric muscle strength (Newton) and muscle composition before and after the intervention. Values are presented as median (25th-75th percentile). % indicates the median relative change following the training intervention.

N, Newton; pQCT, peripheral quantitative computed tomography; CSA, cross-sectional area.

*p*: *p*-value from within-group analyses (Wilcoxon signed rank test), *p*_baseline_: denotes p-value from between-group comparisons at baseline (Mann-Whitney *U* test), *p*%: *p*-value from between-group comparisons of percentage change (Mann-Whitney *U* test). **Bold** values denote *p* < 0.05.

Post intervention, within-group analysis showed that both patients and controls increased in isometric elbow flexion, with large effect estimates (Table 3, Fig. 1A–C). Between-group analysis revealed no difference in the size of isometric muscle strength improvement. No other improvements were observed.

**Fig. 1:**
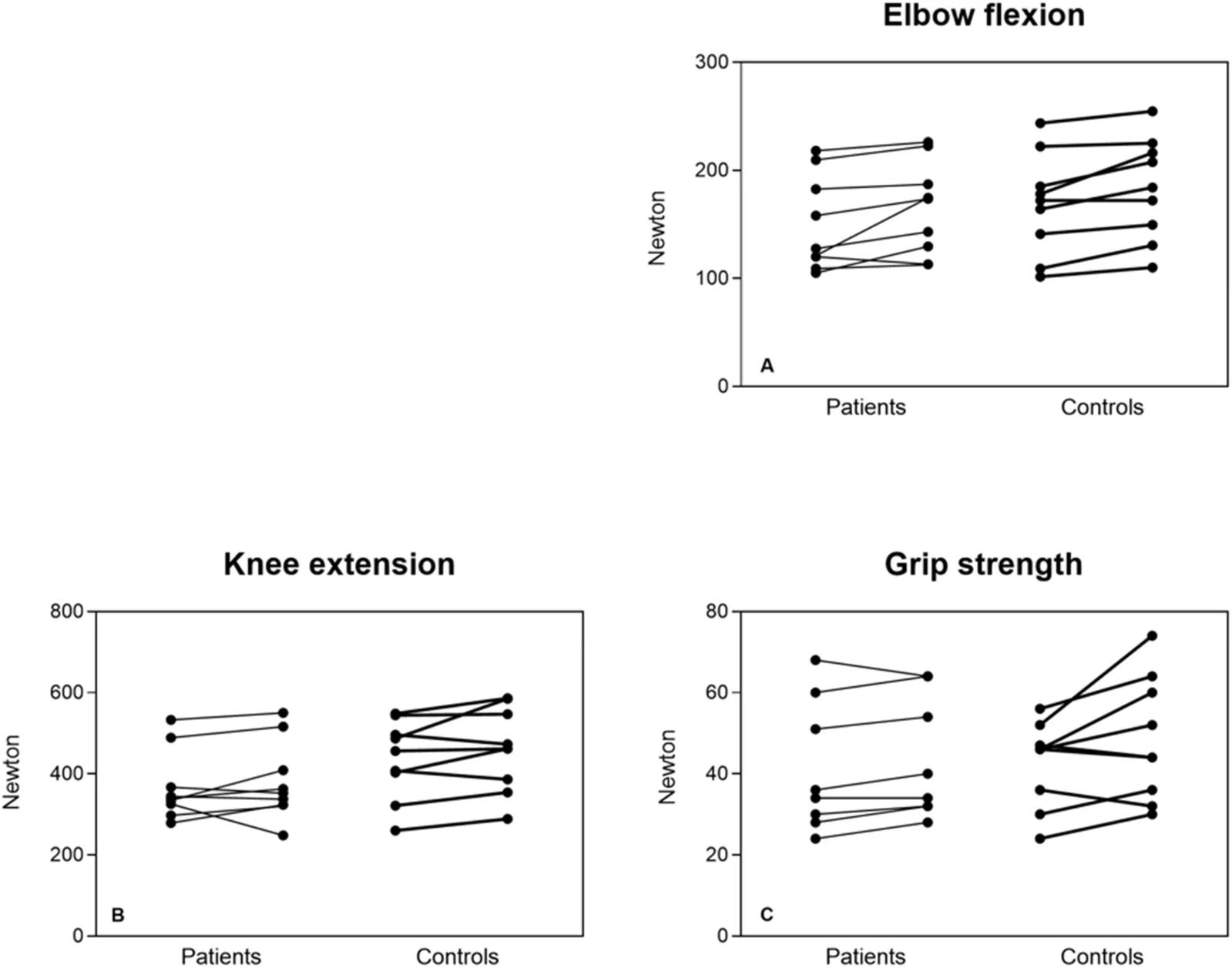
A-C. Isometric muscle strength *pre* and *post* intervention Isometric muscle strength (Newton) in A) elbow flexion, B) knee extension and C) grip strength for patients and controls *pre* and *post* intervention.

### 3.3 Muscle CSA and muscle density

At baseline, radial and tibial muscle cross-sectional area and muscle density were similar in patients and controls (Table 3). Following the intervention, both groups showed an increase in tibial muscle cross-sectional area. Additionally, patients showed increases in muscle density in both the radial and tibial sites. The effect size estimates represented large effects for all three measures. However, between-group comparisons revealed no differences in the magnitude of these changes between patients and controls (Table 3).

### 3.4 Habitual physical activity

#### 3.4.1 Self-reported data

At baseline, both patients and controls reported a habitual physical activity level corresponding to moderate levels. Following the intervention, only patients self-reported physical activity increased with a median score corresponding to high levels of habitual physical activity. However, between-group analysis revealed no difference between the groups regarding the magnitude of changes following the intervention (Table 4).

**Table 4:** Habitual physical activity before and after the intervention.

	Patients (n = 9)				Controls (n = 9)				Patients vs. controls	
Self-reported PA	*Pre*	*Post*	%	*p*	*Pre*	*Post*	*%*	*p*	*p*_baseline_	*p*%
IPAQ *MET*	1194.0 (655.5-2818.5)	4293.0 (1865.3-4730.0)	97.8 (32.3-308.1)	**0.008**	1365.5 (525.5-3498.8)	2190 (822.8-3747.0)	17.7 (−4.9-280.7)	0.3	1.0	0.3

	Patients				Controls				Patients vs. Controls	
Accelerometer-based PA	*Pre* (*n* = 9)	*Post* (*n* = 8)	% (*n* = 8)	*p (n = 8)*	*Pre* (*n* = 9*)*	*Post* (*n* = 8)	*%* (*n = 8*)	*p (n = 8)*	*p*_baseline_	*p*%

Steps *n*	8446.2 (6404.8-11555.0)	9499.7 (5773.0-10304.4)	−2.4 (−22.8, 16.8)	0.7	10092.8 (7310.4-12588.1)	8593.5 (6566.6-12443.8)	−11.0 (−26.7, 19.9)	0.7	0.6	0.7
Sleep interval *min/d*	463.1 (432.8-580.7)	466.0 (420.8-588.1)	−1.2 (−15.3, 12.1)	0.6	469.9 (423.6-530.0)	457.1 (426.9-557.6)	−1.6 (−7.6, 11.6)	0.95	0.9	0.9
MVPA *min/d*	64.2 (42.7-87.9)	72.5 (40.1-83.3)	−8.8 (−21.9, 18.2)	0.8	79.4 (53.0-92.9)	61.0 (44.5-91.1)	−8.1 (−26.7, 3.2)	0.6	0.7	0.7
LIPA *min/d*	78.6 (63.6-107.8)	66.1 (50.4-123.7)	−9.2 (−22.7, 30.35)	0.6	61.8 (55.2-100.0)	75.3 (65.5-100.8)	−10.8 (−16.9, 43.9)	0.8	0.5	1.0
SB *min/d*	586.8 (573.1-669.7)	699.2 (594.8-710.25)	6.0 (−8.8-20.8)	0.5	664.4 (535.9-702.95)	608.1 (569.7-697.1)	0.8 (−13.1-18.8)	0.95	0.7	0.9

Values are presented as the median (25th-75th percentile). % indicates the median relative change following the training intervention. Self-reported physical activity was assessed using the International Physical Activity Questionnaire (IPAQ) and is reported as a continuous score expressed as metabolic equivalent (MET) minutes per week. Accelerometer-based physical activity was derived from ActivPAL3 and includes total daily step count, sleep interval, time spent in moderate-to vigorous physical activity (MVPA), light-intensity ambulatory activity (LIPA), and sedentary behaviour (SB).

PA, physical activity; min/d, minutes per day.

*p*: *p*-value from within-group analyses (Wilcoxon signed rank test), *p*_baseline_: *p*-value from between-group comparisons at baseline (Mann-Whitney *U* test), *p*%: *p*-value from between-group comparisons of percentage change (Mann-Whitney *U* test). **Bold** values denote *p* < 0.05.

#### 3.4.2 Accelerometer-based

At baseline, no differences were observed in total number of steps, sleep interval, time spent at moderate-to-vigorous physical activity level, light-intensity ambulatory physical activity, or sedentary behaviour between patients and controls. Following the intervention, these variables remained unchanged (Table 4).

## 4 Discussion

This study shows that strength training improves muscle strength and muscle density in the upper limb and muscle cross-sectional area and muscle density in the lower limb of adults with a Fontan circulation. Also, patients self-reported an increased habitual activity following the intervention. Yet, for accelerometer measured activity, no changes were observed. Importantly, no adverse events occurred.

### 4.1 Isometric muscle strength

In this study, we demonstrate that strength training enhances isometric strength in elbow flexion in adults with Fontan circulation, at levels comparable to controls. Furthermore, the effect size estimates indicate clinically meaningful improvements of muscle strength. In contrast, no effect was observed in isometric knee extension or grip strength. This discrepancy may reflect the nature of the training protocol, primarily focusing on movements that likely contributed to greater improvements in dynamic muscle strength [13]. Consequently, the transfer of training effects to isometric strength may therefore have been limited. The lack of improvements in grip strength aligns with previous observations in children with Fontan circulation [25]. Yet, this finding is somewhat unexpected, as most upper-body exercises with use of equipment such as cable machines or dumbbells require isometric handgrip engagement.

Functionally, the lower extremities are more frequently engaged in daily activities such as walking and cycling, whereas the upper extremities are comparatively less utilised. This may render the upper limb musculature more responsive to targeted strength training, potentially explaining the observed gains in elbow flexion strength. Notably, a similar pattern was observed in the control group, with improvements limited to elbow flexion strength. In future research involving evaluation of dynamic strength training, we recommend focusing on evaluation of dynamic muscle strength.

### 4.2 Muscle cross-sectional area and muscle density

Following the intervention, muscle density increased in both the upper and lower limbs. A higher muscle density reflects reduced fat infiltration within the muscle, indicating improved muscle quality. Moreover, muscle density is positively correlated with muscle strength [17]. In addition, the increased tibial cross-sectional area, indicating muscle hypertrophy, underscores the positive effects of the intervention on the lower limb, observed as improved calf muscle endurance [13]. The percentual increase in tibial cross-sectional area was at comparable levels to previously reported increase in calf lean mass in adults with Fontan circulation [11]. Although radial cross-sectional area and grip strength did not improve, the increase in radial muscle density reflects beneficial effects on the forearm as well. To provide a more comprehensive understanding of the response to strength training, we recommend including assessment of muscle mass and density alongside measurements of muscle strength in future research.

### 4.3 Habitual physical activity

Self-reported physical activity increased in the patient group during the week following the intervention, whereas no such changes was observed in the controls. This may indicate a short-term carryover effect of the training program among patients. However, by two weeks post-intervention, device-measured physical activity remained unchanged in both groups, demonstrating that the intervention did not translate into sustained increases in daily activity levels. These findings are consistent with previously reported data: a systematic review of training interventions in individuals with Fontan circulation reported similar patterns, with most studies reporting no short-term change in habitual physical activity [10]. Nevertheless, one study within that review reported increased activity levels one year after the intervention, suggesting that structured training may promote long-term behavioural adaptations in some individuals. Important, in the present study, both patients and controls exhibited improved overall muscle function following the intervention, despite the absence of measurable changes in habitual physical activity.

### 4.4 Study design and adherence

Adherence to the training protocol among patients was moderate, despite the intervention being provided at a gym near participants’ home to improve accessibility. A systematic review reported that improvements in peak oxygen uptake were more common among individuals with Fontan circulation who participated in supervised interventions compared with unsupervised programs [10]. However, these studies primarily involved mixed cohorts of children and adults and focused on aerobic exercise, limiting direct comparisons. Notably, one study comparing hospital-based (supervised) and home-based (unsupervised) training in children with Fontan circulation found no difference in aerobic exercise capacity between groups [26]. Whether supervised training is superior to unsupervised training remains unclear and warrants further investigation.

Although patient adherence in our study was suboptimal, unsupervised training may promote self-confidence and support long-term exercise compliance. Comparable adherence was observed in a supervised hospital-based strength training intervention for adults with Fontan circulation [11], indicating that supervision alone may not improve adherence. Patients with complex CHD often experience reduced exercise confidence [27]; therefore, a supervised introduction followed by a structured unsupervised program could enhance confidence and promote sustained exercise behaviour. Unsupervised training might also offer greater long-term sustainability by encouraging independence in a gym setting. Future studies should determine whether supervised and unsupervised approaches yield comparable outcomes in strength, adherence, and long-term effects.

### 4.5 Limitations

There are some limitations in this report that needs to be addressed. First, the small sample size may explain the absence of baseline differences in muscle strength and soft tissue between patients and controls, findings that have been reported previously [13]. Secondly, technical issues resulted in missing data for pQCT and accelerometer assessments, however, not in a systematic way as indicated by sensitivity analysis. Moreover, the sample included both men and women, leading to variability in data and thereby hampering statistical power, since muscle strength and soft tissue differ by sex.

Self-reported and accelerometer-based measures of habitual physical activity covered two separate weeks, limiting direct comparison. This was due to logistical limitations. Nevertheless, accelerometer and self-reported assessments covered two weeks in total, providing a broader view of the activity over time.

Despite these limitations, improvements in muscle strength and soft tissue were observed, providing valuable insights into the effects of strength training in this unique population.

### 4.6 Conclusion

The results of this pilot study show that strength training is safe and yields clinically meaningful gains in upper-limb muscle strength and muscle composition across both the upper and lower limbs among adults with a Fontan circulation. However, further research is warranted to confirm these findings.

## CRediT authorship contribution statement

**Anna Wikner:** Writing – review & editing, Writing – original draft, Methodology, Funding acquisition, Formal analysis, Data curation, Conceptualization. **Frida Bergman:** Writing – review & editing, Methodology, Investigation, Formal analysis, Data curation. **Daniel Rinnström:** Writing – review & editing, Methodology. **Karna Johansson:** Writing – review & editing, Methodology. **Johan Ljungberg:** Writing – review & editing, Methodology. **Bengt Johansson:** Writing – review & editing, Methodology, Funding acquisition, Conceptualization. **Camilla Sandberg:** Writing – review & editing, Writing – original draft, Methodology, Investigation, Funding acquisition, Formal analysis, Data curation, Conceptualization.

## Ethical standards

The authors assert that all procedures contributing to this work comply with the Helsinki Declaration of 1975, as revised in 2008, and has been approved by the Swedish Ethical Review Authority (2022-00058-01, 2022-04769-02).

## Declaration of generative AI and AI-assisted technologies in the writing process

During the preparation of this work the authors used *Microsoft 365 Copilot* ([Large language model], Microsoft Corporation, Redmond, US) in the writing process to improve language and readability of the manuscript. After using this tool, the authors reviewed and edited the content as needed and takes full responsibility for the content of the published article.

## Financial support

The work was supported by the 10.13039/501100003793Swedish Heart-Lung Foundation , Sweden (20230593), the Swedish Heartchild Foundation , Sweden (3/21 Fo, 14/22 Fo), the 10.13039/100018870Swedish Heart and Lung Association , Sweden (Fa 2021-14), the 10.13039/100002129Heart Foundation of Northern Sweden , Sweden, and the County of Västerbotten, Sweden (RV-1014317, RV-1035192)

## Declaration of competing interest

The authors declare no conflicts of interest.
